# Anatomical relationship between the vinculum breve derived from the flexor digitorum superficialis tendon and the volar plate in the proximal interphalangeal joint of the hand: variation in the distribution of attachments

**DOI:** 10.1007/s12565-025-00887-6

**Published:** 2025-07-23

**Authors:** Takeo Ichigaya, Keiko Fujita, Tomohiro Kurisaki, Kazuhiro Takano, Masabumi Nagashima

**Affiliations:** Department of Anatomy, Faculty of Medicine, Saitama Medical University, 38 Morohongo, Moroyama-Machi, Iruma-Gun, Saitama, 350-0495 Japan

**Keywords:** Anatomy, Cadaver, Proximal interphalangeal joint, Vinculum breve, Volar plate

## Abstract

The vinculum breve, a structure connected to the flexor digitorum superficialis tendon, located near the volar plate of the proximal interphalangeal joint. It has traditionally been regarded as a conduit for vascular supply to the tendon and its sheath. However, the detailed morphology of the vinculum breve and its relationship to the volar plate have not been fully elucidated. In this study, we examined the vinculum breve at the proximal interphalangeal joints from the index to little fingers of 20 cadavers (160 fingers in total). Two morphological types of the vinculum breve were identified: cord-like (59.4%) and membranous (40.6%). The membranous vinculum breve attached to the proximal interphalangeal joint tended to be denser proximally than distally. A high proportion (82.5%) of the vinculum breve was found to be attached to the volar plate. Cord-like vinculum breve was significantly more common in the index and middle fingers, where it attached to portions of the volar plate. In contrast, membranous vinculum breve was significantly more frequent in the little finger, often covering a large portion of the volar plate. These findings suggest that during flexion of the proximal interphalangeal joint, the vinculum breve contributes to the proximal sliding of the volar plate in coordination with the flexor digitorum superficialis tendon.

## Introduction

On the palmar side of the fingers, two types of vincula are connected to the tendons of the flexor digitorum superficialis (FDS) and flexor digitorum profundus (FDP): the vinculum breve (short vinculum, VB; plural: *vincula brevia*) and the vinculum longum (long vinculum, VL; plural: *vincula longa*) (Standring 2021). These structures are remnants of the mesotendons. The VB connected to the FDS tendon from the index to little fingers is located near the proximal interphalangeal (PIP) joint and contributes to the blood supply of the FDS and FDP tendons, flexor tendon sheath, and volar plate (VP) (Armenta and Fisher 1984; Beckham and Greenlee 1975). The morphology of the VB has been illustrated in textbooks and prior studies (Bowers et al. 1980; Shao et al. 1995). Gupta and Tamai (2021) described the VB as having a conical shape near the PIP joint; however, detailed anatomical descriptions remain limited.

**Fig. 1:**
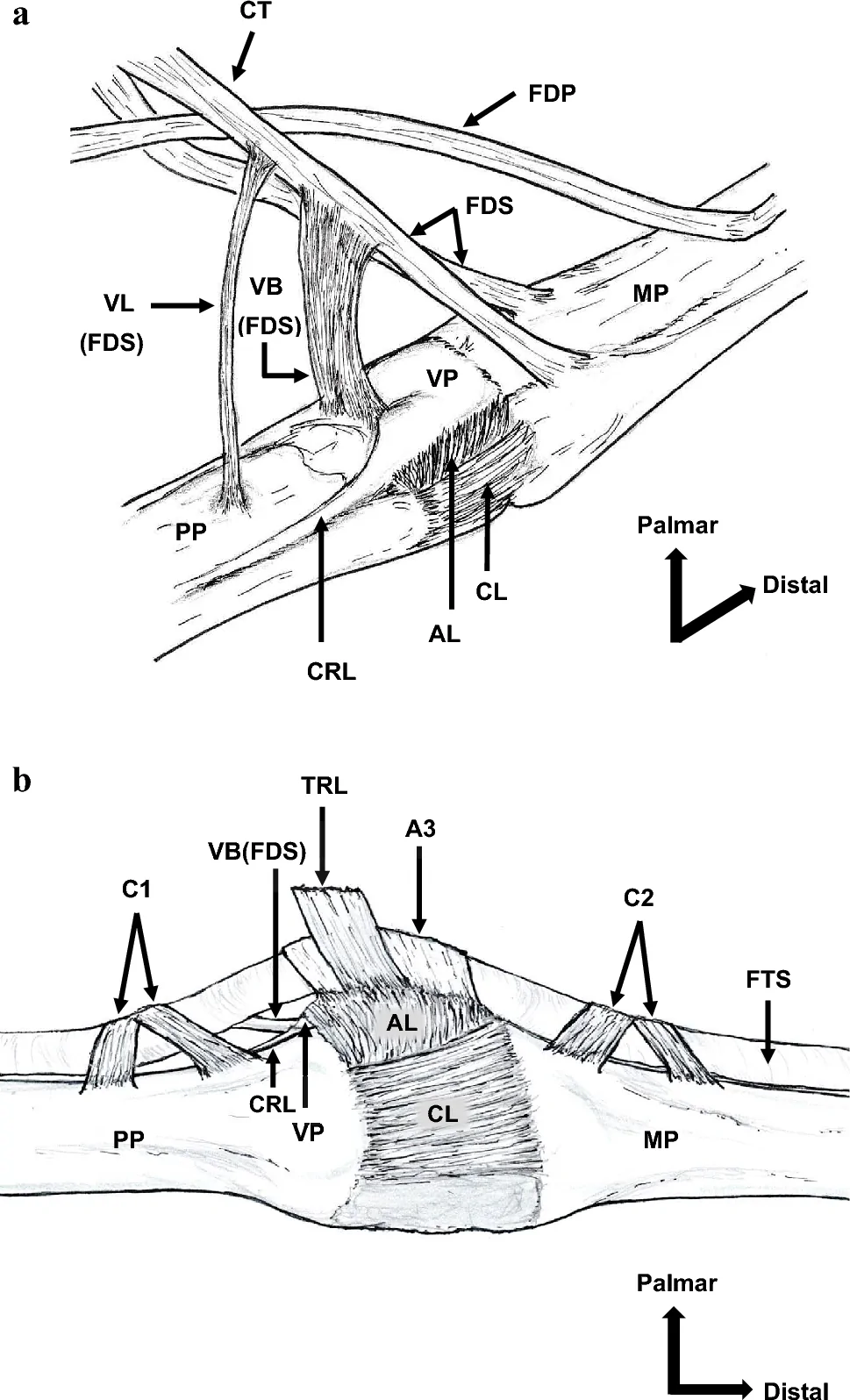
External appearance of proximal interphalangeal joint (schematic diagram). **a** Appearance from the palmar proximal side. **b** Appearance from the lateral side following transection of the TRL. AL: accessory ligament, A3: A3 flexor tendon sheath (A3 pulley), CL: collateral ligament, CRL: check-rein ligament, CT: chiasma tendinum, C1: cruciate 1 pulley of flexor tendon sheath, C2: cruciate 2 pulley of flexor tendon sheath, FDP: flexor digitorum profundus, FDS: flexor digitorum superficialis, FTS: Flexor tendon sheath, MP: middle phalanx, PP: proximal phalanx, TRL: transvers retinacular ligament, VB: vinculum breve, VL: vinculum longum, VP: volar plate

The VP is a fibrocartilaginous structure located on the palmar side of the PIP joint, near the attachment site of the VB. Its substantial thickness plays a critical role in preventing hyperextension of the joint during finger extension (Bowers et al. 1980). Moreover, the VP changes shape during PIP joint flexion, facilitating smooth joint motion (Lutter et al. 2023; Saito and Suzuki 2011). Several structures attach to the VP, including the accessory ligament (AL), A3 pulley of the flexor tendon sheath, transverse retinacular ligament (TRL), flexor tendon sheath (FTS), and check-rein ligament (CRL). All these structures contribute to the stability and mobility of the finger (Bowers et al. 1980; Doyle 2001; Lutter 2023) (see Fig. 1). Although the VB has been reported to partially cover the VP (Armenta and Lehrman 1980), its detailed morphology and anatomical relationship with the VP remain unclear.

As mentioned above, the VB has traditionally been regarded primarily as a conduit for vascular supply. However, a previous report by Sasaki and Nomura (1987) described cases in which PIP joint flexion remained possible after flexor tendon injury, likely due to traction from the attached VB. Based on these observations, we hypothesize that the VB also interacts with the VP and contributes to PIP joint motion. The aim of this study is to clarify the morphology of the VB connected to the FDS tendon and elucidate its anatomical relationship with the VP at the PIP joint.

## Materials and methods

### Cadaver specimens

In the present study, we used 20 cadavers (11 males, 9 females; age range, 62–92 years; mean age, 80.35 years) donated to Saitama Medical University for education and medical research. The PIP joints of the second to fifth fingers were examined (80 fingers on the right hand and 80 fingers on the left hand, totaling 160 fingers). None of the 160 fingers included in this study exhibited ligamentous injuries, osseous deformities, or any history of surgical intervention at the PIP joint. The cadavers were fixed in 10% formalin. This study received approval from the Saitama Medical University Ethics Committee (approval number: Dai2023-021). Furthermore, this work conformed to the values laid in the Declaration of Helsinki (1964). The authors hereby confirm that every effort was made to comply with all local and international ethical guidelines and laws concerning the use of human cadaveric donors in anatomical research.

### Morphological examination

The second to fifth fingers on both hands were incised from the palmar side of the PIP joint. The annular part of the fibrous sheath, known as the A3 pulley, which covers the FDP and FDS tendons and the FTS, was also incised. The FDP tendon was carefully extracted from the tendinous chiasm after dissection. Following this procedure, the FDS tendon was dissected from its insertion at the MP, and the attachment of the VB, which connects the FDS tendon to the VP, was macroscopically examined. The VP is anatomically continuous with the volar joint capsule at the PIP joint; however, in this study, its proximal edge was defined as the region where a distinct and abrupt increase in tissue thickness was observed. This boundary was used to differentiate the VP from the adjacent, thinner joint capsule during gross anatomical inspection. Based on the VB attachment pattern to the VP, the VB was classified into three types: type I, where it did not attach to the VP; type II, where it attached to < 50% of the VP surface area; and type III, where it attached to > 50% of the VP surface area.

### Statistical analysis

In the statistical analysis, we compared the morphology of the VB and its attachment to the VP across the index to little fingers. A chi-squared test was performed, and the Benjamini–Hochberg procedure was applied to control the false discovery rate. Statistical significance was set at p < 0.05. Statistical analysis was performed using js-STAR XR 1.9.6.j. (https://www.kisnet.or.jp/nappa/software/star/).

## Results

### Morphology of the VB

**Fig. 2:**
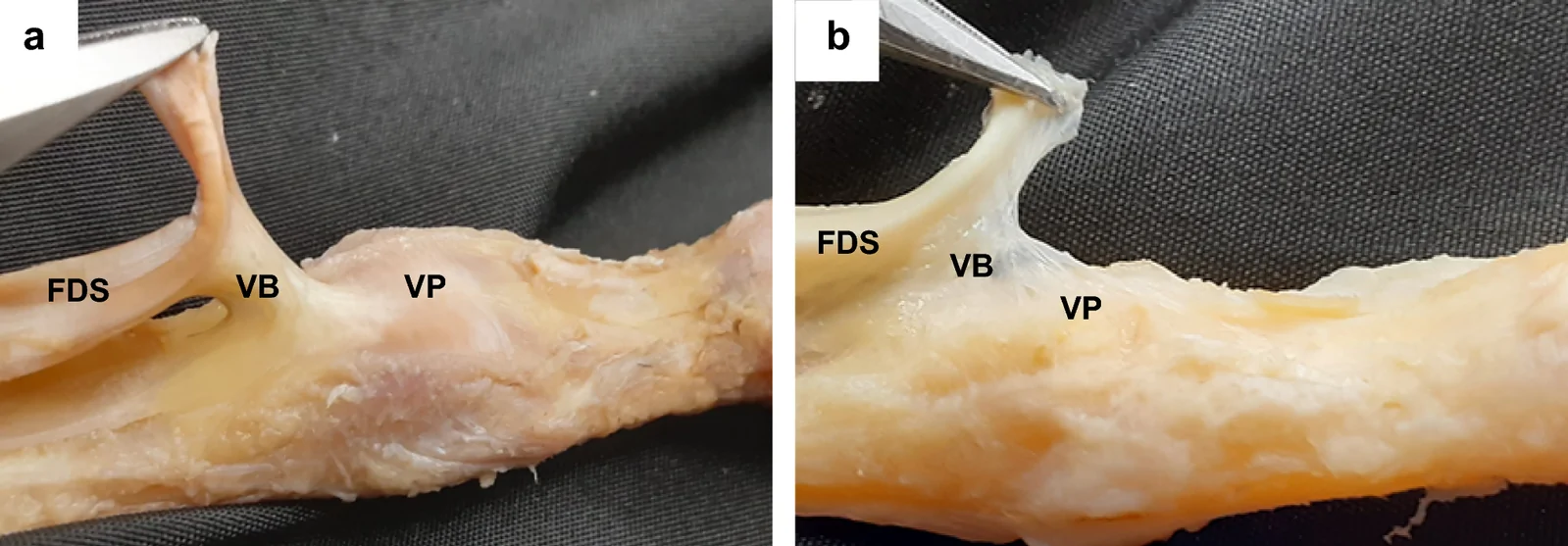
Morphological types of vinculum breve: cord-like and membranous. **a** Cord-like type. **b** Membranous type: The membranous VB appeared denser proximally and sparser distally. FDS: flexor digitorum superficialis, VB: vinculum breve, VP: volar plate

**Fig. 3:**
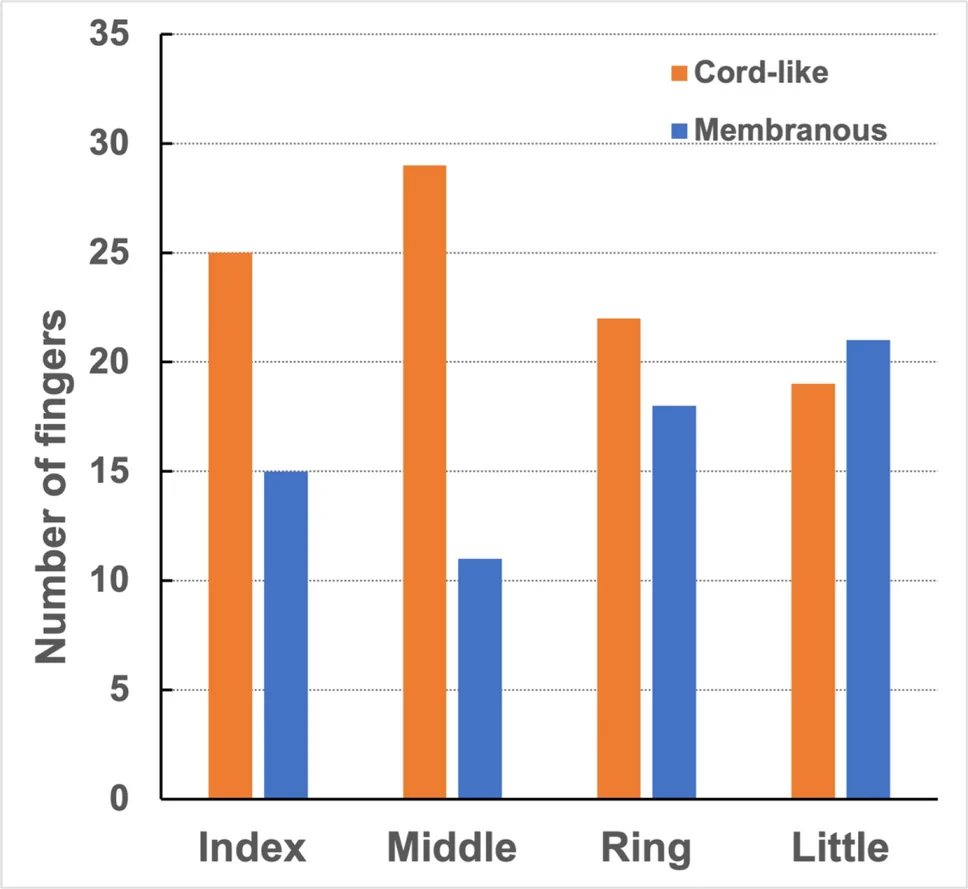
Distribution of vinculum breve types by finger. Bar graph showing the distribution of cord-like and membranous vinculum breve types in the index, middle, ring, and little fingers (n = 40 per finger). No significant differences were observed among the fingers

Macroscopic examination of the VB connected to the FDS tendon at the PIP joints from the index to little fingers revealed two distinct morphologies: cord-like and membranous (Fig. 2a, b). A cord-like VB was observed in 95 of 160 fingers (59.4%), whereas a membranous VB was observed in 65 fingers (40.6%) (Table 1). A comparative overview of these morphologies is presented in Fig. 3.

**Table 1:** Distribution of cord-like and membranous vinculum breve types in each finger (n = 40 per finger)

Fingers	Cord-like type				Membranous type			
	R	L	Subtotal	Total	R	L	Subtotal	Total
	*n*	*n*	*n*	*n* (%)	*n*	*n*	*n*	*n* (%)
Index	12	13	25	95 (59.4)	8	7	15	65 (40.6)
Middle	16	13	29		4	7	11	
Ring	11	11	22		9	9	18	
Little	9	10	19		11	10	21

The VB was attached to the volar aspect of the PIP joint, including the joint capsule, surrounding soft tissues, and VP.

The membranous VB appeared denser proximally and progressively sparser distally. The extent of the membranous portion varied, with some specimens extending to the middle phalanx. Cord-like VB accompanied by membranous expansions was classified as membranous.

No significant differences were found in the frequencies of cord-like and membranous VB among the fingers.

### Distribution of the VB attachment to the VP

**Fig. 4:**
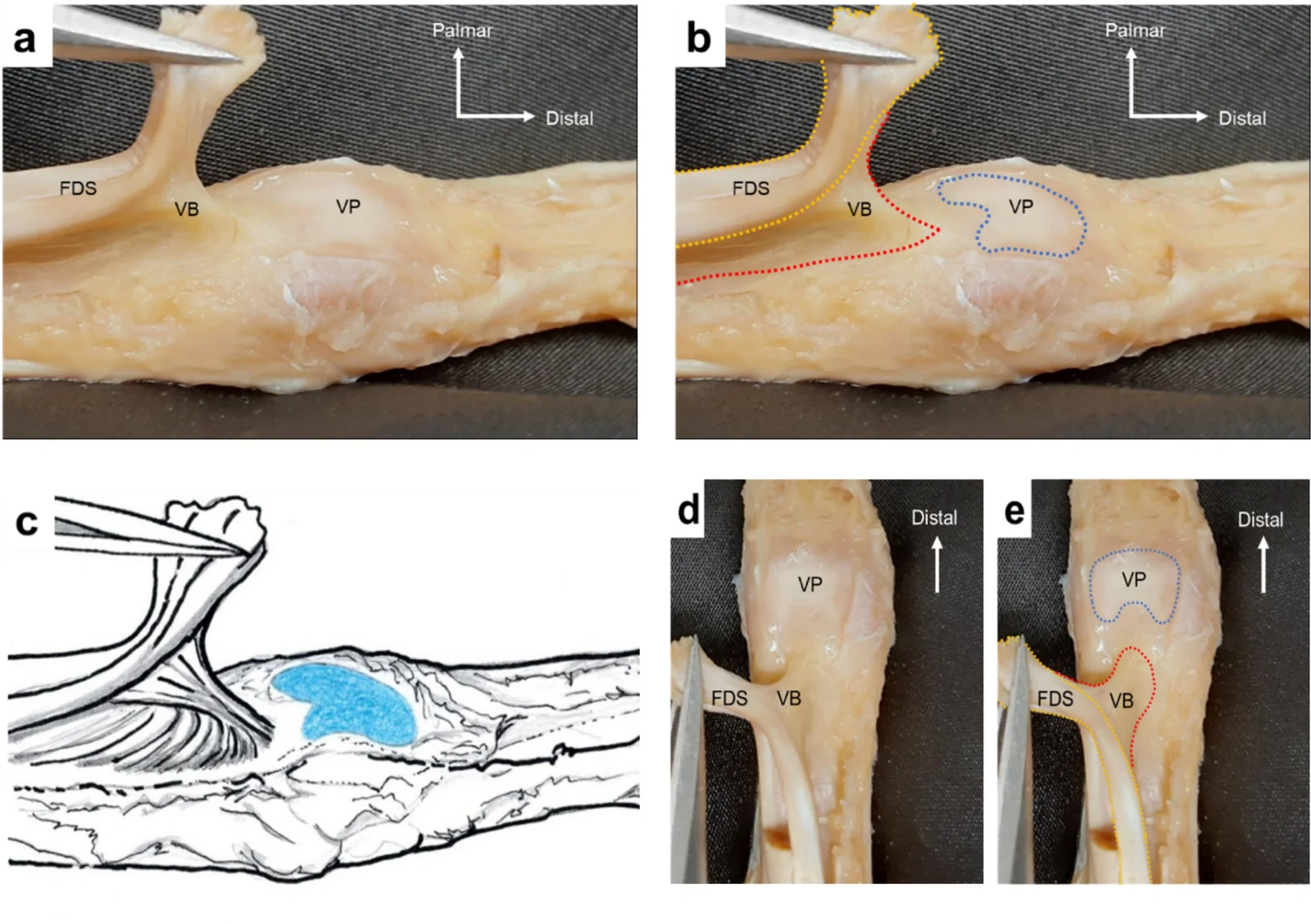
Type I. **a** Appearance of type I seen from lateral and oblique directions. **b** Yellow dotted line (FDS tendon); red dotted line (VB); blue dotted line (VP). **c** Appearance of type I seen from palmar and oblique directions (illustration). *Blue indicates VP. **d** Appearance of type I seen from the palmar direction. **e** Yellow dotted line (FDS tendon); red dotted line (VB); blue dotted line (VP). FDS: flexor digitorum superficialis, VB: vinculum breve, VP: volar plate

**Fig. 5:**
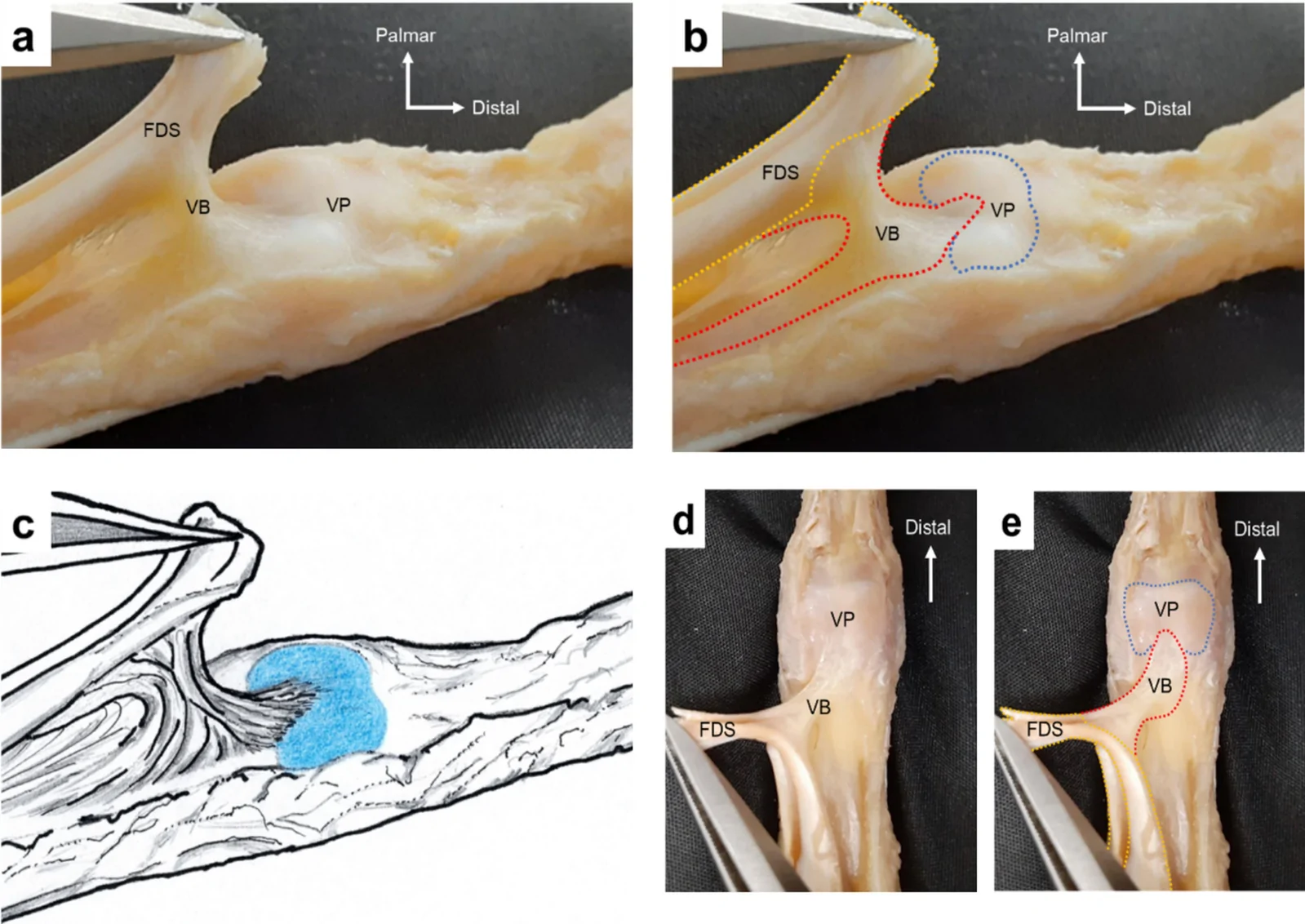
Type II. **a** Appearance of type II seen from lateral and oblique directions. **b** Yellow dotted line (FDS tendon); red dotted line (VB); blue dotted line (VP). **c** Appearance of type II seen from palmar and oblique directions (illustration). *Blue indicates VP. **d** Appearance of type II seen from the palmar direction. **e** Yellow dotted line (FDS tendon); red dotted line (VB); blue dotted line (VP). FDS: flexor digitorum superficialis, VB: vinculum breve, VP: volar plate

**Fig. 6:**
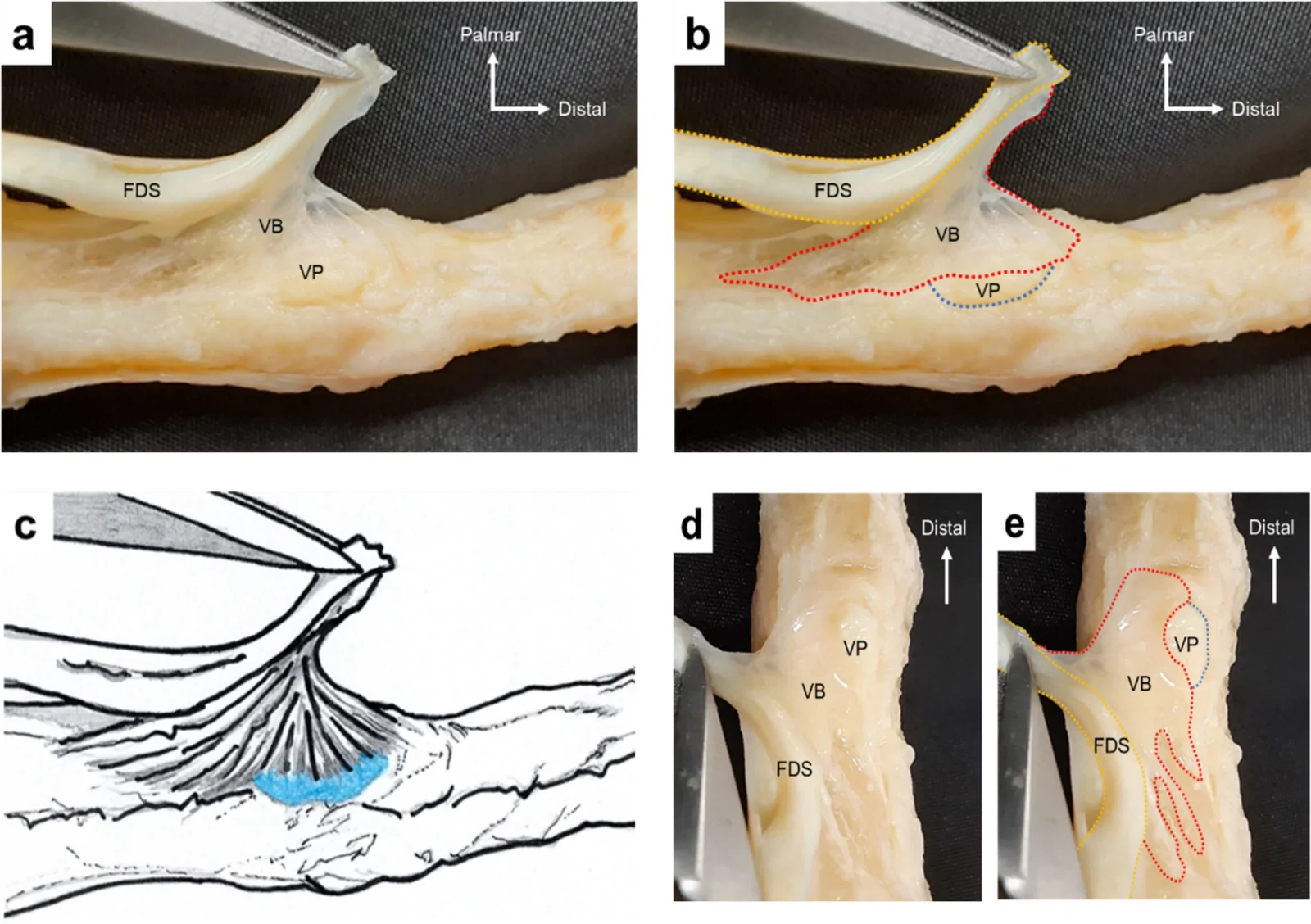
Type III. **a** Appearance of type III from lateral and oblique directions. **b** Yellow dotted line (FDS tendon); red dotted line (VB); blue dotted line (VP). **c** Appearance of type III from palmar and oblique directions (illustration). *Blue indicates VP. **d** Appearance of type III from the palmar direction. **e** Yellow dotted line (FDS tendon); red dotted line (VB); blue dotted line (VP). FDS: flexor digitorum superficialis, VB: vinculum breve, VP: volar plate

The distribution of the VB attachment to the VP was investigated, revealing a wide range of variation—from no attachment to coverage of the entire VP. Based on these findings, the patterns were classified into three types: type I (Fig. 4), in which the VB is attached solely to the joint capsule and surrounding connective tissues without contact with the VP; type II (Fig. 5), in which the VB is attached to less than 50% of the VP surface area; and type III (Fig. 6), in which the VB is attached to more than 50% of the VP surface area.

Among the 160 fingers examined, type I was observed in 28 cases, with 18 on the right side and 10 on the left side, accounting for 17.5% of all fingers. Type II was identified in 68 cases, with 37 on the right side and 31 on the left side, representing 42.5% of all fingers. Type III was identified in 64 cases, with 25 on the right side and 39 on the left side, constituting 40.0% of all fingers. A summary of these distributions is presented in Table 2.

**Table 2:** Distribution of vinculum breve attachment types in each finger (n = 40 per finger)

Fingers	Type I				Type II				Type III			
	R	L	Subtotal	Total	R	L	Subtotal	Total	R	L	Subtotal	Total
	*n*	*n*	*n*	*n* (%)	*n*	*n*	*n*	*n* (%)	*n*	*n*	*n*	*n* (%)
Index	4	2	6	28 (17.5)	9	10	19	68 (42.5)	7	8	15	64 (40.0)
Middle	4	1	5		13	11	24		3	8	11	
Ring	6	3	9		7	6	13		7	11	18	
Little	4	4	8		8	4	12		8	12	20	
Total	18	10	28		37	31	68		25	39	64	

The percentage of VB attachment to the VP was 82.5%, indicating a high rate of attachment. Additionally, among the 64 cases classified as type III, 25 occurred on the right side and 39 on the left, with a significantly higher frequency on the left (p < 0.05) (Table 2).

**Fig. 7:**
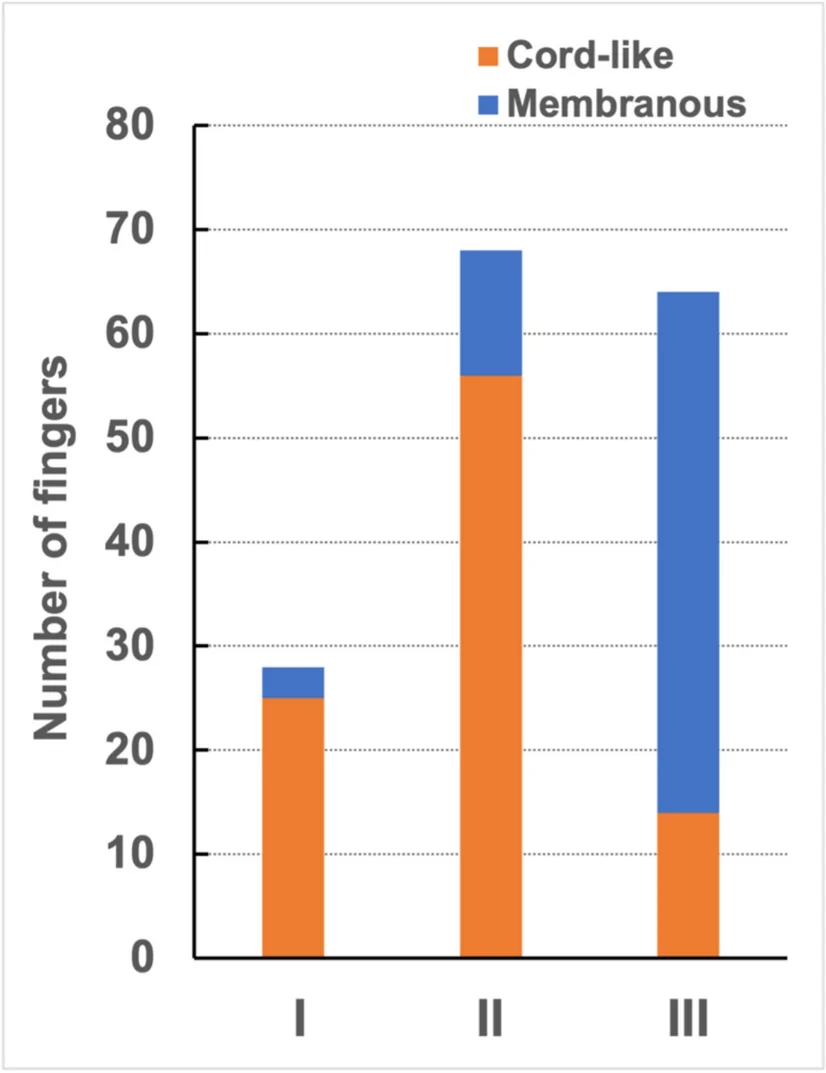
Distribution of vinculum breve morphology by volar plate attachment type. Stacked bar graph showing the number of fingers exhibiting cord-like (orange) and membranous (blue) types of the vinculum breve according to volar plate attachment types. Cord-like vincula brevia were significantly more common in types I and II (p < 0.01), whereas membranous vincula brevia were significantly more frequent in type III (p < 0.01)

We also examined the morphology of the VB in relation to its VP attachment type (Table 3, Fig. 7). Cord-like VB were significantly more common in types I and II (p < 0.01), whereas membranous VB were significantly more frequent in type III (p < 0.01).

**Table 3:** Vinculum breve morphology (cord-like or membranous) according to attachment types

	Type I	Type II	Type III
Cord-like Membranous	25 3	56 12	14 50

**Fig. 8:**
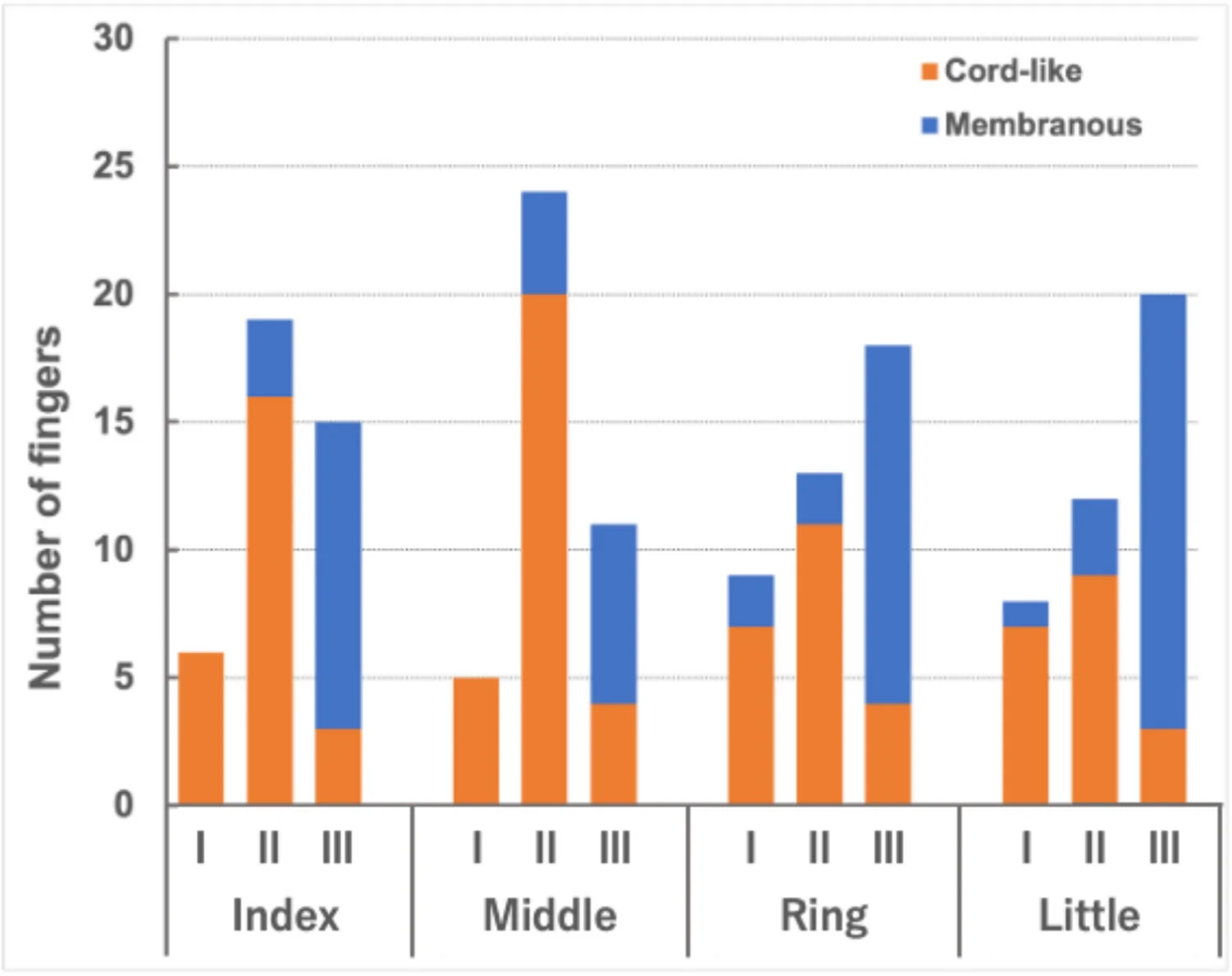
Distribution of volar plate attachment types and vinculum breve morphology by finger. Stacked bar graph showing the number of fingers classified by volar plate attachment type (I–III) in each finger (index, middle, ring, and little), further divided into cord-like (orange) and membranous (blue) vinculum breve morphologies. Type II was significantly more common in the index and middle fingers, while type III was more frequently observed in the little finger. No significant difference was found in the ring finger

When analyzing type distribution by finger, type II was significantly more common in the index and middle fingers, while type III tended to be more common in the little fingers. No significant difference was observed between types in the ring finger (Fig. 8).

Finally, we compared the VB attachment type and morphology across the index to little fingers (Fig. 8). Type II, characterized by a cord-like VB partially attached to the VP, was predominant in the index and middle fingers, whereas type III, featuring a membranous VB broadly covering the VP, was more frequently observed in the little finger.

## Discussion

Although the VB has traditionally been regarded as a conduit for vascular supply, we hypothesized that it also contributes to the PIP joint motion in coordination with the VP. Therefore, we investigated the morphology of the VB connected to the FDS tendon and its anatomical relationship with the VP at the PIP joint.

The VB connected to the FDS tendon has been illustrated as a triangular structure near the PIP joint (Standring 2021), and Gupta and Tamai (2021) reported that it arises from the proximal phalanx and forms a conical shape between the CRLs. Nonetheless, detailed morphological descriptions remain limited.

In this study, cord-like VB (59.4%) and membranous VB (40.6%) were observed. Both types showed a high rate of attachment to the VP. The distal portion of the membranous VB exhibited variable morphology, ranging from termination near the proximal VP to extension toward the middle phalanx. Given that the membranous VB was denser proximally and sparser distally, it is presumed that the distal portion of a cord-like VB may expand into a membranous structure.

The VB has been reported to be as firm as suture thread, with tension in the VB and displacement of the VP observed during PIP joint flexion (Güler and McGrouther 1992; Stewart et al. 2007); however, the specific attachment sites of the VB have not been clearly described. Saito et al. (2012) reported that, based on ultrasound observations, the displacement of the VP during PIP joint flexion occurs in three phases: in phase 1, the VP is tractioned proximally with the sliding of the FDS tendon, and the inclination of the condyle of proximal phalanx (PP) (distal end of the PP) begins to move in parallel; in phase 2, when the angle of flexion exceeds 30 degrees, the most distal part of the VP bulges toward the palmar side, protruding and increasing in thickness; and in phase 3, the most distal part of the VP protrudes toward the palmar side of the MP surface if the flexion angle exceeds 45 degrees, while the MP rotates dorsally within the recess (Saito and Suzuki 2011). Additionally, they reported that the A3 trochlea transmits the tension of the FDS and FDP tendons, protruding the VP in the palmar direction; however, they did not mention the specific structures involved in the proximal traction of the VP in phase 1.

**Fig. 9:**
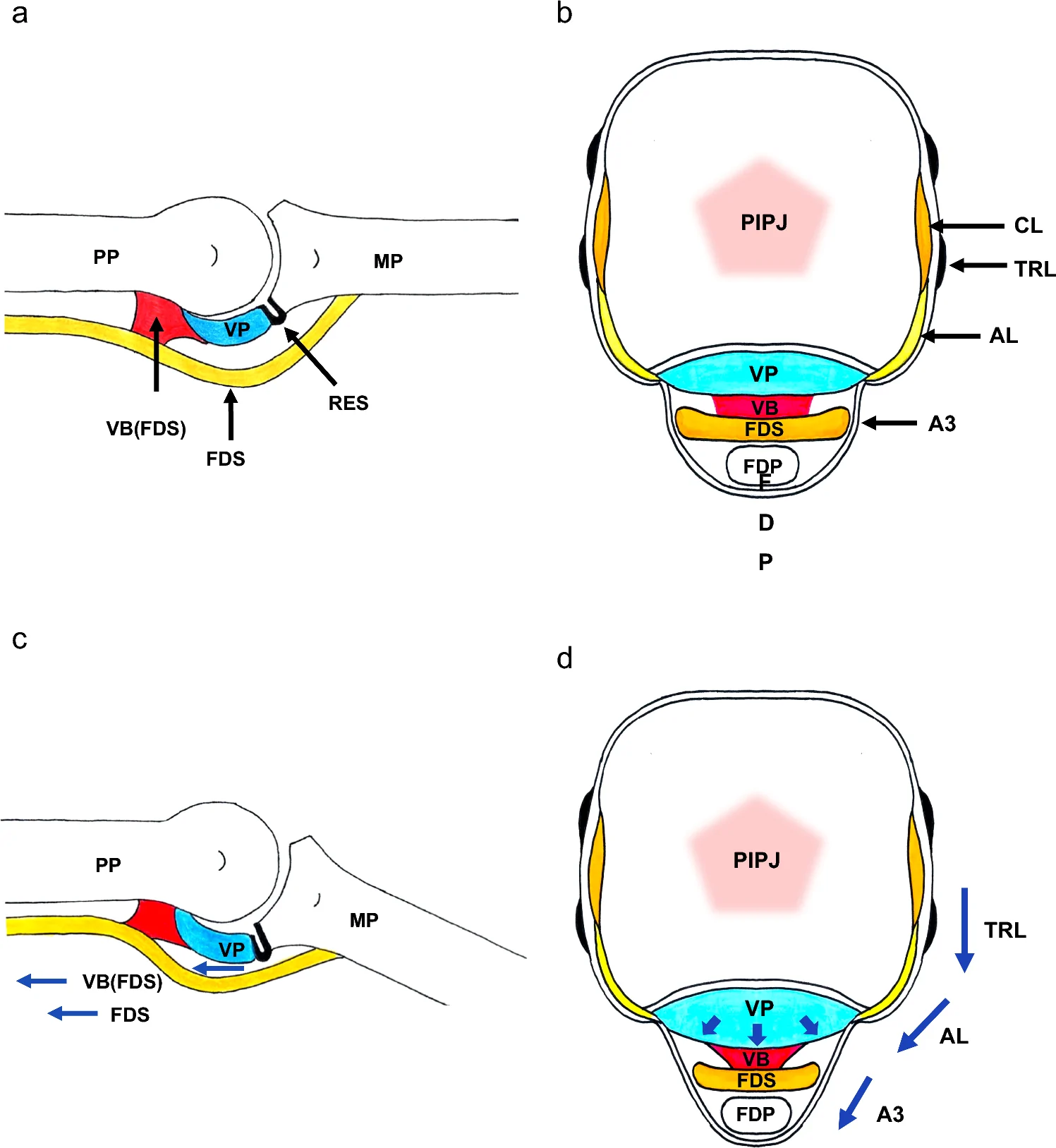
Volar plate and major tissues involved in movement during proximal interphalangeal joint extension. To clearly demonstrate the attachment of the VB to the VP, the CRL has been omitted. **a** Side of the PIP joint (neutral position with neither flexion nor extension). **b** Horizontal plane of the PIP joint (neutral position with neither flexion nor extension). **c** Side of the PIP joint (flexion 0 to < 30°). As the FDS tendon slides proximally during the early flexion of the PIP joint, the VB pulls the VP proximally (the CRL is also involved at the same time). **d** Horizontal plane of the PIP joint (flexion 30° and above). As the PIP joint flexes further, the TRL and A3 trochlea move in the palmar direction. The middle and proximal portions of the AL barely change in length and assist the movement of the VP. AL: accessory ligament, A3: A3 pulley, CL: collateral ligament, FDP: flexor digitorum profundus, FDS: flexor digitorum superficialis, MP: middle phalanx, PIPJ: proximal interphalangeal joint, PP: proximal phalanx, RES: recess, TRL: transverse retinacular ligament, VB: vinculum breve, VP: volar plate

Our macroscopic observations revealed that the superficial layer of the VB was continuous with the VP (Type II, III). These findings suggest that the VB facilitates smooth PIP joint flexion by inducing proximal translation of the VP during the initial phase of joint motion (Fig. 9).

Tissues attached to the VP include the AL, A3 pulley, TRL, FTS, and CRL (Bowers et al. 1980; Doyle 2001; Lutter 2023) (Figs. 1, 9). Particularly, the fibers of the AL exhibit mobility and are tense when the PIP joint is extended and relaxed when flexed (Pai et al. 2019; Chen et al. 2015). These findings suggest that the AL, which prevents excessive tension during PIP joint flexion, may assist in the proximal traction of the VP mediated by the VB (Fig. 9).

Although the VP is anatomically continuous with the volar joint capsule at the PIP joint, it exhibits fibrocartilaginous features and a notable increase in thickness relative to the adjacent capsular tissue. In this study, we operationally defined the proximal edge of the VP as the point where a distinct and abrupt increase in tissue thickness was observed, and we used this criterion consistently during macroscopic examination. Even in type I, where the VB does not directly attach to the VP, its distal attachment eventually connects to the VP via the joint capsule of the PIP joint; therefore, it cannot be concluded that the VB is entirely uninvolved in the morphological changes of the VP.

**Fig. 10:**
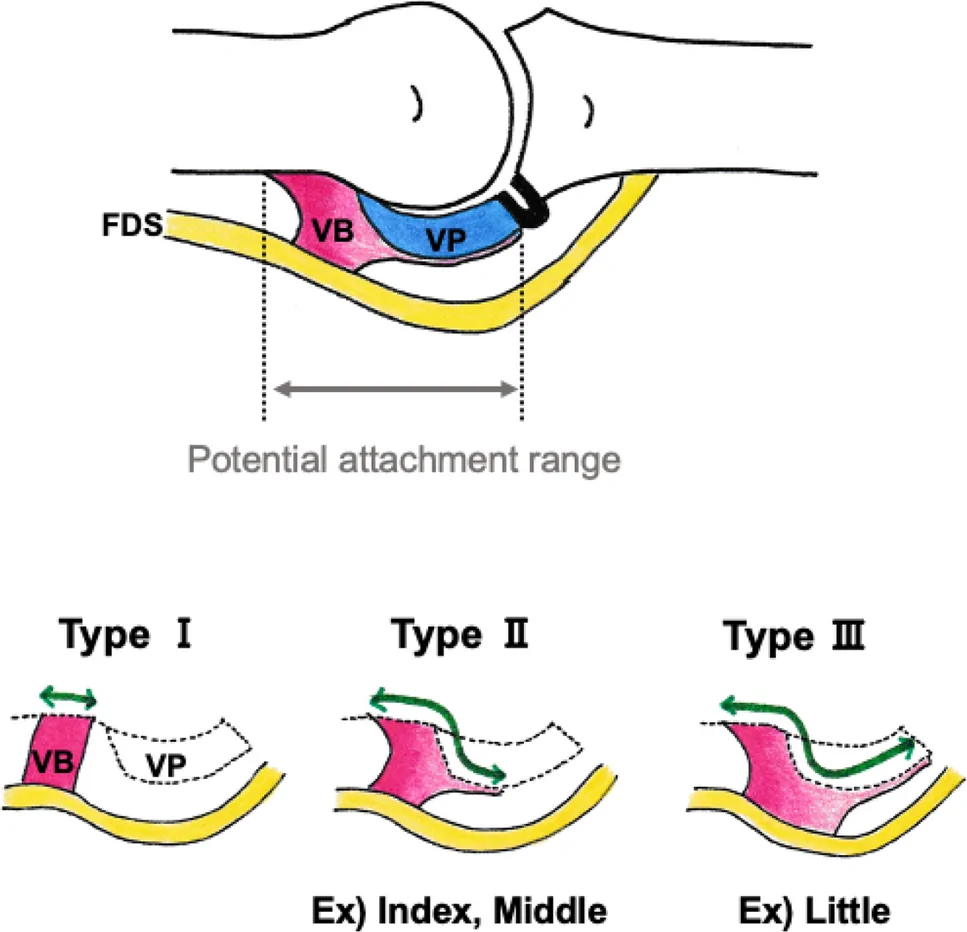
Schematic diagram of vinculum breve attachment types and their functional implications. Diagram showing the lateral view of the PIP joint (top) and the three types of VB attachment to the VP (bottom). During the early phase of PIP joint flexion, contraction of the FDS translates the VP proximally via the VB. Type I: The VB indirectly translates to the VP through the joint capsule. Type II: The VB translates the VP directly. Type III: The VB broadly attaches to the VP, compensating for the weak FDS strength of the little finger and enabling more efficient proximal translation of the VP. The green arrows indicate the attachment area of the VB to the VP. The membranous part of the VB is dense proximally and becomes sparser distally. VB: vinculum breve, VP: volar plate, FDS: flexor digitorum superficialis

Regarding finger-specific differences in attachment type, type II was more frequently observed in the index and middle fingers. In contrast, type III was more commonly observed in the little finger. The FDS muscle strength in the middle finger is reportedly 75% greater than that in the index or ring finger, while that in the little finger is approximately 50% lower (Brand and Hollister 1999). The predominance of type II in the longer fingers, such as the index and middle fingers, may reflect the sufficiency of partial VP attachment in generating effective proximal translation of the VP. The cross-sectional area of the FDS tendon in the little finger is significantly smaller than in the other fingers (Baker et al 1981). Additionally, Kömürcü et al. (2008) described the VP of the little finger as being thin in the central and lateral parts. Considering the functional weakness of the little finger compared to the other fingers, the extensive attachment of the VB to the VP observed in type III, may help maintain the flexion efficiency of the PIP joint in the little finger (Fig. 10).

Avulsion injuries at the VP attachment site on the middle phalanx have been reported in cases of PIP joint hyperextension injuries involving the index to little fingers (Eaton and Littler 1976; Paschos et al. 2014).

The anatomical and functional observations regarding the VB and its relationship to the VP presented in this study may provide basic insights into the pathophysiology of such injuries, by suggesting how tension transmitted via the VB could influence stress distribution around the VP region.

To fully elucidate the roles of the VB and VP in finger biomechanics, future studies should investigate the mechanical effects of the VB on the VP in vivo. 

## Data Availability

The data that support the findings of this study are available from the corresponding author upon reasonable request.
