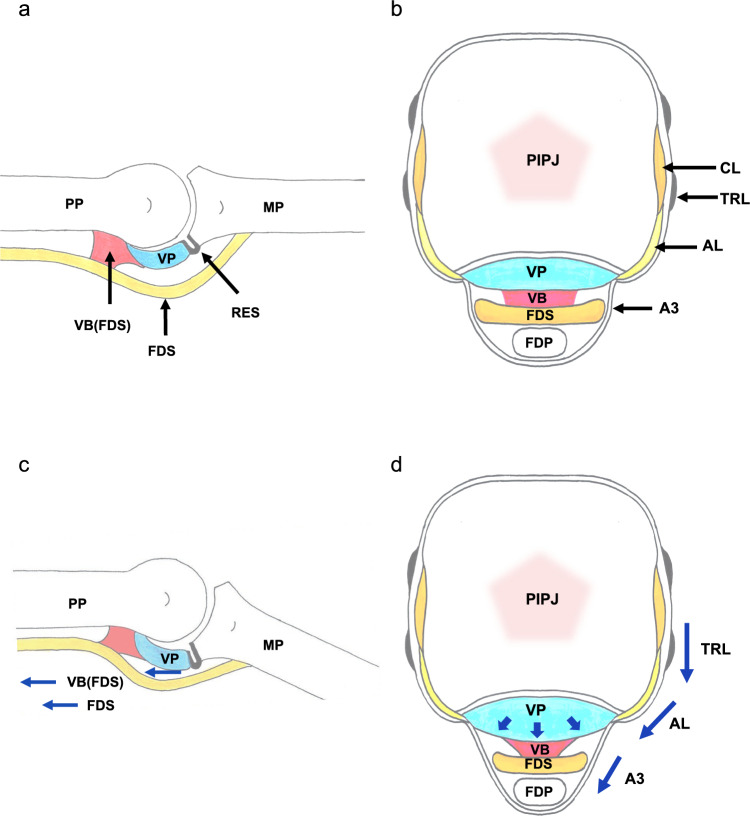# Correction: Anatomical relationship between the vinculum breve derived from the flexor digitorum superficialis tendon and the volar plate in the proximal interphalangeal joint of the hand: variation in the distribution of attachments

**DOI:** 10.1007/s12565-025-00894-7

**Published:** 2025-08-26

**Authors:** Takeo Ichigaya, Keiko Fujita, Tomohiro Kurisaki, Kazuhiro Takano, Masabumi Nagashima

**Affiliations:** Department of Anatomy, Faculty of Medicine, Saitama Medical University, 38 Morohongo, Moroyama-Machi, Iruma-Gun, Saitama, 350-0495 Japan

**Correction: Anatomical Science International** 10.1007/s12565-025-00887-6

In Fig. 9b of this article an unintended text string (“FDP”) appears vertically in the central area beneath the PIPJ; but should have appeared as in this correction.